# Caring for the world: Geography, Religious Cosmovision and Encounters, Elizabeth Wilson’s ‘actionist’ career, 1943-1990’

**DOI:** 10.30827/dynamis.v44i1.30727

**Published:** 2024-06-30

**Authors:** Bertrand Taithe

**Affiliations:** University of Manchester

**Keywords:** care, humanitarianism, religion, spiritualism, Elizabeth Wilson

## Abstract

This paper focuses on the spatial imaginings of a pacifist, developmentalist humanitarian, and poly-activist who was engaged from the Second World War onwards with refugees, immigrants in the 1960s, development projects in the 1960s and 1970s, pacifism during the Vietnam war, and antinuclear campaigns of the Campaign for Nuclear Disarmament. Elizabeth Wilson presented the peculiarity of being immersed in developing a spiritual notion of care. Through her engagement with Buddhism, Zen, and Hinduism, as well as her pursuit of psychoanalytical collective unconscious based on the work of Carl Jung, Elizabeth Wilson offers an original perspective of what it meant to be a humanitarian at a crucial juncture in the 1960s. This paper will attempt to reconcile the specificity of her activism with the wider narrative of internationalism in the 1960s and 1970s by paying attention to how she related her often fragile sense of self with her role as a carer connected to broader and deeper networks of inspirational (female) figures. While she denied being a feminist and preferred to describe herself as a human being (also highlighting a maternalist identity by repeatedly mentioning she was a ‘mother of four’), Elizabeth Wilson built friendships and ‘actionist’ (their word) networks of care that helped her ‘sustain’ her idealism and pursue her ‘journey’. Elizabeth Wilson undertook internationalist causes as a spiritual quest and a personal self-construction, and her writings, diaries, photographs, and rich personal archives enable us to approach the concept of care in the after-war as a central concern in the development of a new humanitarian consciousness.

## 1 Introduction

Molly Andrews in her pathbreaking exploration of elderly socialists ^1^ recalled her unusual encounters with an odd, elderly, socialist-humanitarian-pacifist, Buddhist, Quaker woman from Huddersfield. Whereas she struggled to make Elizabeth Wilson fit with the more conventional accounts of class consciousness or political awakening she had gathered in a book largely concerned with class identity, she rightly emphasized Elizabeth’s independence. The old middle-class woman had characteristically narrated her singular life journey through the account of one of her most often repeated dream narratives. In this story which served as a parable for her life to date, she described travelling in a dinghy alongside a crowded pleasure cruise ^2^. She, alone in her small one-person ship, was looking in awe at the great bulk of a cruise ship sailing by. The activist —who used the neologism ‘actionist’ to denote her view on direct action— emphasized from the onset her engagement with notions of active caring about, caring for and being open to receiving care in her interaction with the world ^3^.

In other interviews and in her own writing, Elizabeth Wilson (1909-2000), emphasized the role played by spirituality and religion in her life long career as a humanitarian ^4^. Through Unitarianism, a very liberal religious group of a few thousand members which denies the trinity and preaches a very open approach to God ^5^, Andrews quoted, Elizabeth had found ‘greater spiritual understanding of respect for spirituality in anybody, the whole world over…’ ^6^. Elizabeth’s self-narratives which dominated the final decade of her life, the period during which Andrews interviewed her, brought coherence and consistency to a life which had at times appeared a curious mix of adventure, travels and more conventional provincial lifestyle. Yet through a singularly rich and intimate archive we can approach in depth the intimate thoughts and ideas of an original woman who was credited by Matthew Anderson of being one of the earliest British founders of ethical fair trade in the modern era ^7^. Elizabeth Wilson set up a local NGO and collaborated with Oxfam throughout her life; she campaigned in pacifist and Quaker circles in the UK and abroad ^8^. She travelled from 1960 onwards and documented major humanitarian needs; she served as a conduit for a wider awareness of ‘third world’ issues. While Andrews drew on Marxist notions of commitment to investigate these unconventional elderly militants —Elizabeth reflected on notions of care and spiritual fellowship which built on the therapy she had received as a patient to establish the ground for a cosmology of care.

Throughout her life Elizabeth cared. This caring persona was defined by her positionality as a woman, mother and human being. She particularly forefronted her maternal identity in all the confrontations she had with the justice system in the course of her pacifist activism ^9^. While she never claimed to be influenced by feminism, her lifelong interest in women spiritual leaders, particularly Christian Mystics in her early years and later Buddhist and Zen nuns, framed how she herself engaged with other activists. Through her notebooks, memoirs and writing we can engage with the cosmology and spiritual geography underpinning her notions of care. As a figure whose overseas activism began in earnest when most contemplate the prospect of retirement Elizabeth Wilson crossed a number of key periods in the history of humanitarian response —from wartime activism to the 1980s. Her profile was revelatory of the sort of domestic humanitarianism Liisa Malkki explored for Finland’s women who contribute to humanitarian causes through their arts and craft and domestic volunteering ^10^. Her international activism completed and reinforced local activism and networks in which she cut an unusual space for herself which transcended her original gender and class role as a middle class married woman.

## 2 Wilson, class and care

Elizabeth Wilson came from a moderately privileged background, she studied geography at university, met a PhD student in Chemistry at a Unitarian church in Cambridge, married in 1936 and moved to aa industrial town —Huddersfield— during the great depression. Married teachers were not expected to work, and raising a family became her primary social obligation ^11^. Elizabeth Wilson had four children, three boys and one girl, and defined herself first and foremost as a mother throughout her life. The couple moved to a city which would have been socially quite alien to them. She noted in her biography that until the war made it a relatively safe haven, southern friends and relatives could not fathom this move. The couple made their life in this town dominated by chemistry (ICI), engineering and textile industries. Elizabeth’s husband joined the labour party and was elected in the 1960s to the town’s council. Elizabeth and him were very active in religious networks, and as the war began, in pacifist activism ^12^.

As a middle-class educated woman new to the region, the notion of care she was expected to embrace was that of social care. West Yorkshire had been very affected by the depression and Elizabeth Wilson took on to let part of her house to pauper families at a preferential rate. She followed a friend in participating in social work at the Family Service Units in Oldham, another industrial town across the Pennine hills ^13^. Almost on arrival in Huddersfield she originally got involved in soup kitchens providing for the more destitute. From soup kitchen to international relief —as James Vernon would see it—there was only one step in the imagined governance of poverty relief ^14^. During the war her relief work took two distinct directions: pacifist activism in favour of war prisoners and caring for Jewish refugees in her own home. In this she fitted with the specific British strand of pacifism inspired by Quakerism and Unitarianism. This work was combined with meetings and engaging socially around questions of peace building. As Adam Millar showed in his thesis ^15^, Elizabeth set up a specific organisation to fundraise for the victims of the Bengal famine in 1943 ^16^. There were then myriad organisations set up across Britain, though many, like the Oxford Famine Committee, were really responding to the Athens famine caused by the British Navy blockade. To respond to famines associated with Imperial policies was to contest the British government in the midst of the war. Hudfam was set up as a famine relief committee like Oxfam —the two organisations were the only ones which survived the decades following the war. Oxfam became by far the most successful of the two and, when the founders’ generation of Hudfam retired, took over its activities in Huddersfield.

In the 1990sElizabeth Wilson, described Hudfam as being concerned with the ‘different standards of living in the world’ ^17^. This late analysis of Hudfam belonged firmly to a perspective that had developed in the 1980s and is not that clearly identifiable in the archives. Famine relief in the 1940s has to be understood as a short-term fundraising exercise rather than an institution building enterprise. In the wider history of humanitarianism these short-lived social responses tend to leave the least imprint in any archives. The study of Hudfam and the work of Elizabeth Wilson more widely opens a windowe on what was often a temporary efflorescence of compassion during a period of almost universal devastation and potential inuring to sufferings. The fund-raising activities: meals, flag days sanctioned by local authorities and dinners on behalf of the starving were all opportunities for the strengthening of social charitable networks which, as sociologists of charitable work have shown, constructed the awareness of their cause as they established the legitimacy of action. Where relief funds differ from what later became non-governmental organisations was in the remit of these activities. Fundraisers tended to dispatch resources to identified brokers or local organisations —something Hudfam did throughout its existence— utilising missionaries, Quaker organisations, and local activists as the medium for doing good. Until 1960 Hudfam relied on such networks to deliver the bounty gathered by the local network of activists —gifts in kind (knitted blankets and recycled items of clothing) and money. Whereas the original organisation also relied on these organisations to report back and demonstrate the effectiveness of the relief work undertaken in Hudfam’s name, Elizabeth Wilson added, through her self-funded visits, another layer of documenting. Elizabeth thus left a body of articles which she wrote abroad —acting as the foreign correspondent of the local Newspaper, the *Huddersfield Daily Examiner—* and photographs which she then bundled into slide shows ^18^. From 1960 she thus toured with these slide shows devoted to the rescue work undertaken in Agadir, work with refugees in Palestine in 1967, work in Bangladesh in 1972, Lebanon in 1976 or with more geographical themes devoted to India or the far East, Eastern religions etc.

Elizabeth Wilson thus straddled two worlds —one of philanthropic organising and social networking around issues of fundraising— and one of conveying back the state of the world. Her travels were thus exploratory of the state of the planet and human needs charity, but her travel writings also sought to establish connections and clear parallels between social conditions in Asia and the West. While often not clearly delineating the boundaries of the exotic —her narratives were often very specific about flora, fauna, and landscapes— she nevertheless insisted on the common experiences of humanity facing poverty and on the physical and spiritual care needed to enable well-being. Her pre-war work in the community was for her good preparation for what she encountered in the war-torn countries she visited (like South Vietnam in 1973) or developing nations facing problems of chronic poverty such as the Nilgiry Hills of India where her son Jonny worked most of his life. In these travels Elizabeth Wilson relied on a range of organisations and contacts who facilitated her visits but she travelled mostly alone. While her readings and personal encounters in Huddersfield had exposed her to overseas cultures (she housed foreign students of the polytechnic throughout the 1960s until the 1980s) she began her travel in 1960. These regular forays abroad enabled her to combine her interests for geography and tourism and her growing religious cosmological curiosity. Her visits gradually fed into a broader quest for meaning, causalities and connections to locate human sufferings and appreciate what brought humanity together ^19^. Originally her travels were on disaster sites or war zones on behalf of Hudfam (Agadir Earthquake in 1960) but from a visit to the holy land in 1967 onwards she combined holy sites visits and encounters, visiting with Ashrams in India or Buddhist temples in Cambodia in 1968 or Vietnam in 1973. At the difference of those embarking on the self-discovery journey of the hippy trail ^20^, Wilson a woman of over 50 when she began travelling always concerned herself with care work as a way to inform her notions of self.

The paradox of this lone traveller seeking to commune in collective enterprises was marked and her experience of pacifist activism in 1967 and 1968 when she joined a group of British pacifists committed to ending the Vietnam war by pacifist means, was a frustrating experience. While the committee embarked on a peace deputation to Vietnam, it ended up stranded in the sport complex of Phnom Penh, unable to do much more than witness the first evidence of what later became America’s secret war of mass bombing in Cambodia ^21^. Elizabeth stood out from this group and sought to be more action driven and went to Hong Kong to seek direct action opportunities. There she attempted to join the American Quaker ship Phoenix of Hiroshima —which had in previous years delivered aid to North Vietnam and she leafleted American soldiers on leave in Hong Kong ^22^. Elizabeth Wilson had established links in Hongkong since 1960 when she agreed to turn Hudfam into the distributing agent of refugee arts and craft coordinated by the Lutheran refugee council ^23^. This initiative which used a retail catalogue to distribute silk-embroidered objects (notably tea cosies such as the ones kept in her collection) has been regarded as an early instance of fair trade and certainly predated the development of charity shop imports of fair-trade goods. By that stage Hong Kong refugees had been enlisted in a portfolio of handicraft programmes run by all Christian denominations ^24^. By 1960 over 100,000 people were homeless in Hong Kong, 160,000 people arrived in 1962 alone ^25^. Typically, the handicraft enterprise of the Christian organisations in Hong Kong sought to develop a culture of rehabilitation, resettlement and training of refugees to prevent them from developing refugee apathy, the inactive despondency of assisted people without hopes, as analysed by Peter Gatrell ^26^. The production of artisanal craft was thus integral to a therapeutical approach based on empowerment which was meant to develop agency, combined with a desire to see refugees become useful economic agents in their new surroundings ^27^. Developing a trade in these objects was always a difficult secondary objective ^28^ and Wilson’s engagement in setting up a sustainable trade for these artefacts —not on the ground of their intrinsic quality but because their production entailed a narrative of care— was a significant step forward at a time when trading barriers made the import of goods difficult. Purchasing these goods was in itself akin to a form of active support ^29^. Hudfam had a charity shop of its own in Huddersfield but nothing that could match the retail network established by Cecil Jackson Cole for Oxfam and later Help the Aged ^30^.

Elizabeth Wilson’s interest in the trade of goods belonged to her wider concerns with development work. Historians of developmentalism have shown how, in years following the end of empires these concerns became key modes of envisioning the world ^31^. The emphasis on expertise and improvements to modes of production in the countryside has been well studied ^32^. How these concerns could be associated with other ways of relating to sufferings in the world and caring at distance along the lines suggested by John Silk remains to be explored more fully ^33^. For Elizabeth Wilson humanitarian and development work went alongside her pacifism and was informed by it. Retailing the work of refugees was to enable them to earn a way out of their condition and repair some of the sufferings they had incurred as a result of the war in China. Her notions of care as part of a wider activism in humanitarian and development concerns thus related to her sense that inequalities were productive of conflict. But her understanding of caring for the world also entailed a sense of self-reflexion and care for the self.

## 3 Caring for the self

Elizabeth Wilson has left us enough archives to give us a clear idea of her personal motivations in details unavailable to many historians attempting to engage with humanitarian lives ^34^. In the 1950s Elizabeth Wilson clearly experienced a nervous breakdown which probably also combined with a crisis of faith ^35^. Her mother had passed away recently and she began to read more widely in books of theology —paying particular attention to the lives of female mystics such as Hildegard of Bingen or Teresa of Avila but also more broadly began renewing her acquaintance with Eastern religions she had read into in her youth ^36^. Her therapist introduced her to the psychoanalysis of Carl Jung. This enabled Elizabeth Wilson to make more sense of her own mystical experiences, in particular her vivid dreams but it also offered a system of thoughts rooted in comparative theology. Jung had long used religious texts from across the world to establish his notions of collective unconscious.

In Jungian psychoanalysis the collective unconscious could be related to by individuals, and dreams were the key to the register of images and tropes shared by entire humanity. Looking for keys and clues within her own unconscious became a lifelong pursuit for Elizabeth. From her wide-ranging readings in Christian theology and in particular Christian mystics, Elizabeth developed an educated interest in Daoism, Confucianism and Buddhism. Combining this extensive spirituality was not unique to Elizabeth of course and she relied on the many second-hand interpretative sources available to these cultures which had appeared in the 1940s and 50s. Like many hippies of the 1960s, her concerns were not mere curiosity but a desire to find healing through an encounter with the divine or some sort of revelation. In many ways her journey was one of undefined theism or God optional religion as May puts it ^37^. Interestingly, while many of her generation went on that track, she did not draw in Khalil Gibran or Sufi Islam.

Her ‘discovery’ of Hinduism in the 1940s and 50s and Buddhism in the 1960s did not lead to a conversion to any particular sect or religious group finding new disciples in the west ^38^. She remained a Quaker throughout her life but she added to the specific discipline of this intensely self-reflexive religious group the daily practice of Buddhist meditation, Ghandian non-violence, Tibetan dream divination, Zen Buddhism and Ikabena. If this spiritual journey was initially undertaken with a view to self-heal and recover from depression —in order to find a place for herself after the more intense period of familial duties— Wilson also sought to make this healing relate to broader issues. Her pacifism and developmentalism were, to her, directly connected to the encounters her religious quest enabled. For instance, her visit to Vietnam and her discovery of socially engaged Buddhism was a personal encounter too. She admired socially engaged Buddhists not only because of their actual social work and development activities but because of their profound spirituality ^39^. Similarly, her pacifism which originated pre-war in her unitarian faith, was confirmed through her encounter in the 1930s with Gandhi’s writing on nonviolent resistance and reaffirmed through her dual belonging to the CND and Quakerism ^40^. By the time she was arrested for protesting against Polaris missiles in Scotland in 1962, her CND activism was not dominant and she gradually came to the realisation that to show care was to be directly involved and take part. The travels to Cambodia and Hong Kong in 1968, ‘to Asia in Peace’ in the peak of the Vietnam war was to confirm her willingness to put herself in the line of fire in the name of peace ^41^.

## 4 Humanitarian cosmovision

Elizabeth Wilson connected every aspect of her existence into a vast rhizomic network of encounters and exchanges. Though she never read Gilles Deleuze and Félix Guattari, she might have thought familiar some of their metaphysical perspectives on networks and on the unconscious ^42^. To recognize these connections for her was not a philosophical endeavour but an empirical approach which entailed a profound appreciation for people and things and a metaphysical understanding of the world. Her writings at the end of her life —at a time when she lost sight in one and then both eyes revealed how she informed her cosmovision or humanitarian cosmology:

Knowing that I was going into hospital I was well prepared and had “in the footsteps of the Buddha” by Rene Grousset ^43^ ready to re-read…the many dangers he [the Chinese Buddhist monk] faced in a spirit reminiscent of a true Quaker is nourishing food for contemplation. This is highly recommended reading, very suitable for those with only one eye in action. But a year later with no sight and five days waiting in hospital before an operation was a more difficult problem to solve. The Tibetan Book of the Dead tells the pilgrim to face the angry ghosts and other architypes [sic] of Tibetan Buddhism and “walk on” so tried to do just that… Memories of yesteryear began to open up the dry channels and gradually I became alive again, a golden thread vibrated, linking experiences people, animals, especially my Sheltie bitches, trees, archetypal dreams into a living network, an inner life that sometimes one neglects on account of other pressures. “For the world is a living web, fragile, subtle, mysterious, linked – Earth and Water, tree and bird are shot through with the glory of god (Fritjof Capra ^44^) ^45^.

It is in a sense typical of her erudite curiosity that, in the same document, specifically addressing how she endeavoured to survive a physical challenge and a closing down of her horizons, Elizabeth Wilson should draw from a wide range of inspirations, ranging from an introduction to Tibetan Buddhist thinking to a recent example of what Sean Watson calls ‘new Bergsonism’ and which he relates to wider philosophies of subjectivity ^46^. Building on Carl Jung’s approach to archetypes to bring into dialogue different religious spiritual belief systems and spiritual exercises ^47^, Wilson could also integrate within her cosmology a range of ‘new age’ philosophical perspectives on transience and interconnectedness of the living. These musings did not take her far from the Gaia hypothesis of James Lovelock and approach a synthesis of rhizomic theories developed by Deleuze and Guattari with east Asian religious practices ^48^. In Wilson’s quest for the divine, archetypes as described by Carl Jung structure religious belief systems and populate the collective unconscious. For Wilson the world is thus made of conscious connections, a web of encounters and happenchance meetings between like-minded souls, and more tightly woven by unconscious connections that can only be revealed through meditating, sudden revelations and deeper thinking. The formal religious practices and the humanitarian undertakings of a Quaker meeting could only go so far for her ^49^, and Wilson combined her Unitarian religious background with an eclectic embrace of Buddhism and Zen Buddhist practices to define herself and shape her social and humanitarian work — what she called ‘care for the world’. Wilson drew on Asian philosophic texts to seek deeper connections. While still a practicing Quaker she deplored the limitation of their thinking: “Our meeting has faithfully done its stint, though I am disappointed that at the end of our studies no sense of Creation Spirituality seemed to emerge” ^50^.

Though Elizabeth Wilson may come across as somewhat idiosyncratic she is by no means unique in the field of humanitarian action. The dominant figure in humanitarian work of the first half of the twentieth century, Dr Albert Schweitzer (1875-1965) who was, since his founding of the Lambaréné hospital in Gabon (1913), the most famous humanitarian in the world was also defined by an extraordinary cosmology combined with a cult of action. In his own words, Schweitzer strove to ‘live [his] thoughts’^51^. Before he was undermined for the reactionary nature of his paternalism or the archaic nature of the medicine he practiced, Schweitzer had been a dominant figure in humanitarian and missionary circles. The downfall was as harsh as the adulation had been strident. In 1963, *Time* magazine published ‘Albert Schweitzer: An Anachronism’, which castigated the man for his apparent ignorance and ‘archaic’ attitude. The focus of this attack and much of the criticism that followed was directed largely at Schweitzer’s ‘obsolete’ medical practices and the sub-standard standards of medical care in his hospital in Lambaréné. However, no aspect of Schweitzer’s life and thought was left unturned. He was criticized for his perceived Communist sympathies —in light of his barbed comments against Western nuclear powers; for his antiquated ‘white-hat colonialism’, some of his paternalist writings, implicit and explicit racism and, in the 1950s, his skeptical approach to African independent states. His liberal critiques also criticized the ambiguities of his Christian belief and for the vague conservative tendencies of his humanitarianism ^52^. By 1975 Brabazon could identify a Schweitzerian ‘myth’ and ‘anti-myth’ which reflected these ambiguities arising from the close scrutiny of his cosmology and practices of care ^53^. In particular, Dr Schweitzer’s creed of ‘reverence for life’, inspired by Jainism had drawn from Asian philosophy to inscribe humanitarian practices in a broader philosophy of respect for all living things. Reverence defined as a ‘poetic concept’, indicative of a solemn acknowledgement before an immense or overwhelming power. It was akin to awe Elizabeth Wilson presented in her writings on the meanings of life ^54^. Where Wilson remained faithful to a psychological and Buddhist approach to life, Schweitzer laid bare the two basic impulses that all living things share that together form the ‘will-to-live’: a desire for self-preservation and a desire for unity with other iving things. The principle of reverence for life serves both impulses insofar as it is founded on the basic premise: ‘I am life which wills to live in the midst of life which wills to live’ ^55^. Schweitzer’s ethic entailed reciprocity, and applied to all life: human, animal and plant. There was a circuitous ‘logic’ to the reverence ethic which entailed that through service one would feel a sense of unity and solidarity, satisfying social needs. In turn, once unity is extended the more others love and serve each other this would support one’s desire for self-preservation ^56^. Schweitzer articulates that when the ‘will-to-live’ begins to think ‘it realizes that it is free to choose whether or not to live’, that is, there is a choice between actualizing one’s authentic existence and failing to do so. Consciousness of this choice, a ‘reverence’ for this choice, leads to the necessity of being sincere with one self ^57^. While Schweitzer maintained some distance between his Christian theology and his philosophy, including in *The Philosophy of Civilization* of 1923, his ethics entailed, like Elizabeth Wilson, a certain distance from canonical orthodoxy and some commentators argued like Clark in 1962 that the ‘ethical mysticism’ of Schweitzer had to be clarified to be regarded as Christian. Drawing attention to the ‘formidable sought ambiguities and apparent contradictions’ in Schweitzer’s thought, Clark seeks to clarify how, for example, he could be both rational and mystical and why he omits reference to God were in some writings whilst meditating on Christ in others. These paradoxes are only superficial for Clark, for he argued that Schweitzer’s understanding of Christian faith is an ‘open minded system in regard to both philosophy and theology’ ^58^. To those who conclude that Schweitzer’s Christianity was dubious and the use of traditional Christian language in his writings disingenuous because he presented his ethical mysticism independently of religious revelation, Clark reminded them that Schweitzer himself spoke of his humanitarian trip to Africa an ‘an act of obedience to Jesus’ ^59^. Whilst Clark observed that Schweitzer derived his concept of the ‘will-to-live’ and the terminology of his Reverence for Life largely from the voluntarists and vitalists of Germany and France, he also highlighted that for Schweitzer, ‘Jesus as the Christ manifests the ‘will-to-love’ which transcends the struggle between creatures ^60^. But the problem Clark perceived is the fact that if Schweitzer is considered to be ‘a legitimate, indeed, a brilliant interpreter of Christian faith’ it will cause ‘surprise, dismay or disgust in various admirers of Schweitzer’, precisely because reception is entirely dependent on where one sits on the spectrum of theology and philosophy: ‘those who regard him as ‘the greatest living Christian’ without any depth of acquaintance with his writings would be shocked to discover how unorthodox he is; those who consider him as a debunker of Christianity will be irritated to find he is firmly entrenched in the Christian tradition’ ^61^. These words could apply very literally to Elizabeth Wilson who added to a shared interest in mystical approach to Christianism, the desire to root its analysis in psychological terms inspired by Carl Jung.

While it may appear contradictory for Wilson to have declared herself a Christian, an agnostic, *and* a Buddhist, recent approaches to Schweitzer also show how one can integrate Schweitzer’s theology and philosophy ^62^. Barsam in a 2008 study explores how Christology can provide the foundation for Schweitzer’s ethical mysticism, that is, his reverence for life ethic should be understood as fundamentally theological.

What this comparison with the most famous humanitarian of the first sixty years of Elizabeth Wilson’s life reveals is that her ethics of care, rooted in networks of deep and intense meaning, was not entirely unique but partook of a wider theological curiosity associated with a cult of ‘humanitarian actionism.’ We thus need to replace her firmly in a socio-cultural context of care grounded in theological education and truth seeking. The humanitarian cosmology to which Elizabeth subscribed and which she defined for herself was profoundly connected to a Christian worldview of charity and compassion, love and care. In her final interview to the local newspaper, in 1998, a newspaper which had followed her travels and work since the 1950s, Wilson could bring out her ecumenical beliefs while also reflecting on the need for better race relations. “Quakers believe that there is ‘that of God’ in everybody. Buddhists believe in the Buddha nature in everyone and Hindus in the *atma* (Spirit) in all people. It is not religion which causes the troubles, but fanatics. Wherever there is love and caring and gentleness, that is like Christianity. People should be loving and compassionate and look for the good in people and build on that” ^63^. What this religious worldview revealed was that care had to be universal in scope and founded in religious ethics. Her personal approach to consumption and recycling, and her advocacy of ecological approaches to agriculture combined with her celebration of communities and collective purpose.

## 5 Geographical reach and development

Ironically for someone who proclaimed the need for collective thinking and action, Elizabeth travelled alone from 1960 until the 1980s. The list of Elizabeth Wilson’s travels on what she described as ‘fact finding missions’ is extensive: Morrocco, Palestine and Israel, India, Cambodia, Bangladesh, South Vietnam, Japan, Nepal, Sri Lanka, Kenya. In an interview of the mid 1980s, Wilson describer her motivations in terms of duty and ethics: “Although she admitted to often feeling terrified and vulnerable on her lone foreign trips, Mrs Willson’s Quaker beliefs gave her the inner strength to keep going. “When I sat down and thought about it, what I was doing was right. I was not idly gawping —I was going to help and plough everything I found back into Oxfam. Even if fell by the wayside it would not have mattered. At least I had tried” ^64^. Wilson took her interest in spatial inequalities to demonstrate the commonality of experiences —often drawing comparisons on poverty in the United Kingdom with the egregious examples she met abroad. What her Quaker obituary in September 2000 called her ‘ministry of teaching and her ministry of doing’ were never separated and one of the main outcomes of these extensive travels was to report back and disseminate images and testimonials through press articles and through a lifelong campaign of public talks across a range of networks, United Nation association, Quaker, pacifists, and women’s groups such as the women’s institutes ^65^.

Her visits included a range of ventures: orphanages in Vietnam, schools in Lebanon and Palestine teaching craft skills, development projects in the Nilgiri hills for the development of local ‘indigenous’ groups, vaccination projects in Cambodia, refugee workshops in Hong Kong or Quaker orphanages in Morrocco. Many of them were concerned with the young and with education. Trained as a teacher in Geography Wilson often referred to pedagogical schemes which, alone, might have the power to heal the world. Insofar as she combined relief work concerns with the urge to empower and educate —to address the root causes of distress rather than simply responding to the symptoms of inequality— Wilson was an exemplary Oxfam militant. As a go-between for local fund-raising events and the delivery of aid in the ‘field’ ^66^ Elizabeth Wilson reflected not only familiar globalisation trends and the newly accessible pull of tourist flights but also a response to demands and meetings she had at home. The globalisation of workforces in the industrial north brought Asian workforces, Arab students, and West Indians to her life. She reflected on the absence of Hinduism in the curriculum after meeting an Indian educationalist in Huddersfield. Her encounters around Buddhism were equally in European and North American contexts and echoed the developments of Buddhism in the west in the 1960s. Her position as a multi activist incredibly well connected to a vast number of groups and causes was that of a catalyst and organiser but it did not necessarily prevent her from being lonely. The individualism which marked her for public notice and made many of the local initiatives revolve around and depend on her was also the source of a deep sense of paradoxical exceptionalism. It is something that is often found as a constant in the writings of humanitarians and carers. Many autobiographical accounts are thus built on similar narratives of happenchance, encounters, and luck —hiding perhaps some self-congratulatory sense of manifest destiny ^67^. The 1969-1970 diaries of one of the founders of Médecins Sans Frontières, Pascal Grellety Bosviel, recounting his quest for God, enlightenment and wisdom in India following a brief stint with the World Health Organisation reflect very similar concerns and cosmovision. While always profoundly doubting his faith and aptitude to grow as a human being, Grellety Bosviel experienced humanity as journey of self-discovery and self-effacement at the same time ^68^. The internal debate between ego-centred worldview and the embrace of the universal was not unique to Wilson but seems to be a recurrent trope of humanitarian ego-histories. In her autobiographical writings and in her journalism Wilson is always very self-aware and wary of exhibiting too much ego — nevertheless she cannot but centre on her self-exploration as the origin of her engagement with the wider cosmic enterprise. Her sense of loneliness is manifest in dreams explored in more depth elsewhere and it amounts to a kind of solipsism — albeit not in a narcissistic meaning of the term but rather as a kind of idealism centred on the self as key to the universal.

## 6 Caring as the connections of all things

Connecting as she did her personal spiritual quest with a constant desire to act, engage and network, Elizabeth did not belong to the secular or purely individualistic evolutions of the 1960s spiritual zeitgeist, yet her universalism depended on this sense of self, albeit a self-connected to the world entire and, through these connections, in search of the divine. Unlike other humanitarians who drew from political perspectives on the holocaust or on socialist internationalism, Wilson developed her own spiritual cosmology. This does not mean however that she did not partake in developmentalist perspectives or that she did not share commonly held views of modernisation. She clearly engaged with these and like Oxfam, more widely subscribed to a developmentalist perspective on agriculture while embracing third wordist views on community. Her spirituality was not so far removed from that of Dr Schweitzer who had been the dominant figure of interwar humanitarianism, his Jainism against her Buddhism ^69^. Arguably, Schweitzer’s notions of care relied on a more medical and pragmatic activism associated with very abstract theological musings while Wilson defined her care through social and spiritual networks supporting people who would be delivering physical care. Her political and social activism was devised around campaigning and fundraising, representing and questioning. Her networks revolved around her own quest, and she never claimed to be a spiritual leader herself, merely a travellers seeking enlightening encounters.

This deeply individualistic spiritualism combined with a profound sense of a duty to care is not unique among humanitarians. Looking more broadly we could find many more instances of the same and it then begs the question of how do we locate her personality among the many thousands of women who did not confide their journeys to diaries or memoirs but nevertheless felt a duty of help —a need to help, to echo Malkki. That this duty remained framed —however open she wanted herself to be— by the constraint of her education remained a source of frustration for her children. Jonny, who lived in India in the Nilgiri mountains, resented the tone and orientalism of her journalism. Her daughter Erica went beyond her in perpetuating her tradition of non-violent activism by being a much more militant feminist pacifist activist.

Elizabeth Wilson’ s caring for the world was framed in religious terms and in a very idiosyncratic philosophy. While we cannot extrapolate to her entire generation the evidence her records provide, we might, nevertheless, posit a few conclusions as to the motivations of humanitarians in the 1960s-1970s. The first point is that the orientalist fascinations which drove many abroad had for some much deeper roots than any superficial desire to travel. The second that the religious context and framing of humanitarian engagement was much more profoundly structuring emotionally, essential to her personal wellbeing and longer lasting than the impressions one might get from the emergence of ‘an NGO moment’, to borrow O’Sullivan’s phrase, dominated by more secularist understandings of internationalism ^70^. The final point is to call for a closer examining of the links between local activists and international causes, paying more attention to the social and emotional networks which sustained international causes like the campaigns against famine — drawing thus on Peter Gatrell’s work on world refugee year ^71^ and Ana Bocking-Welch’s work on hunger campaigns ^72^ and connecting it to the various development and humanitarian causes which emerged and continue to resonate in the manner in which individuals seek to care for the world. That they wished to care for the world is in no doubt — that they needed to and that it also represented a form of self-care is also key to our understanding of their life work. œ

## Footnotes

1 Molly Andrews, *Lifetimes of Commitment, Aging, Politics, Psychology (*Cambridge: Cambridge University Press, 1991), 143.

2 See Bertrand Taithe & Adam Millar, “Living Humanitarian Dreams: The Oneiric and spiritual Life and Activism of Elizabeth Wilson (1909-2000)” *Journal of Cultural and Social History*, https://doi.org/10.1080/14780038.2022.2161806

3 In this she really operated along the lines of care defined by Tronto as highlighted in the introduction to this special issue. Joan C. Tronto, “Du care,” *Revue du MAUSS* 32 (2008): 243-265.

4 Her writings are to be found at the West Yorkshire Archives and in the Humanitarian Archive at the University of Manchester library.

5 Alan Ruston, “British Unitarianism in the Twentieth Century a survey”. *Transactions of the Unitarian Historical Society* 1 (2012): 76-88.

6 Andrews, *Lifetimes*, 130

7 Matthew Anderson, *The History of Fair Trade in Contemporary Britain* (London: Palgrave, 2015) chapter 1.

8 Quakers are a protestant group of singular significance in all manner of social activism see Pink Dandelion, ed. *The Quaker Condition: the sociology of a liberal religion*. (Cambridge: Cambridge Scholars Publishing, 2009).

9 As Dolores Martín-Moruno, Brenda Lynn Edgar & Marie Leyder (2020) Feminist perspectives on the history of humanitarian relief (1870-1945), Medicine, Conflict and Survival, 36:1, 2-18, point out, this compassionate mother identity is often notable among humanitarians., pp. 4-5.

10 Liisa H. Malkki, *The Need to Help: The Domestic Arts of International Humanitarianism*. (Durham, NC: Duke University Press, 2015).

11 This was a social convention rather than the law since the Sex Disqualification Removal Act of 1919.

12 E. Wilson (Undated). Recording of Elizabeth Wilson [Describing the events of her life and Hudfam]. Hudfam Archive, WYAS, KC547/6/7

13 Elizabeth Wilson, *Encounters on the Way* (York: William Session), 8.

14 James Vernon, *Hunger: A modern history*. (Cambridge Mass: Harvard University Press, 2007).

15 Adam Millar, ‘Huddersfield’s Humanitarians: A History of the Aims and Origins of the Huddersfield and District Famine Relief Committee, Master Diss., University of Huddersfield, 2018.

16 Janam Mukherjee, *Hungry Bengal, War Famine and the End of Empire (London: Hurst, 2015*)

17 Andrews, *Lifetime*, p. 131

18 Her articles included: “Aftermath in Agadir”, *Huddersfield Daily Examiner*, 14 December 1961; “They Work to Save Indians in Drought” Article on India written by Elizabeth Wilson (*Huddersfield Daily Examiner*, date unknown?); “Mainichi Daily News Cooperative Farming in India”, *Huddersfield Daily Examiner*, February 1965?; “Scene that Catches at Heart and Leads to World Beyond”, February 15 1965; “I Have Known Poorer People in Huddersfield”, February 26, 1965; “A Leper Colony Run by a Hard Working Idealist”, March 5 1965; “Suddenly the Sun Caught the Peaks of Kanchenjunga”, April 8, 1965; “Lepers Afraid to Enter hospital if They Get There”, March 26 1965; “Many Striking Contrasts in its Old and New Architecture”, April 15, 1965; “Vietnam Self-Help and Rehabilitation”, 16 October 1973. “Towards Self-Sufficiency: New Project with Indian Aboriginal Tribes” (Oxfam publication, no date) Oxfam “Feeding the Needy”, 9 September 1973.

19 Michal Givoni, “Beyond the humanitarian/political divide: Witnessing and the Making of Humanitarian Ethics.” *Journal of Human Rights* 10.1 (2011): 55-75.

20 Sharif Gemie, “Cross-cultural Communication and the Hippy Trail 1957-78.” *Interventions* 19, 5 (2017): 666-676.

21 *Pat Arrowsmith (ed.), To Asia in Peace*: *The Story of Non-Violent Action Mission to Indo-China* (London: Sidgwick and Jackson, 1972).

22 Elizabeth Jelinek Boardman, *The Phoenix Trip: Notes on a Quaker Mission to Haiphong* (Burnsville, N.S.: Celo Press,1985); Tarik W. Kamil, *The Politics of Time and Eternity: Quaker Pacifists and their Activism during the Vietnam War era* (Athens, Ohio: Ohio University, 2006).

23 Laura Madokoro, *Elusive refuge: Chinese migrants in the Cold War*. (Cambridge Ma.: Harvard University Press, 2016).

24 Raymond Earl Clements, “The International Significance of Church-related Relief and Rehabilitation in Hong Kong with Reference to Handicraft Programs”, American University, MA thesis, 1964.

25 L Stumpf, *Report of the Hong Kong Office of the Lutheran World Federation of World Service for the Helsinki Assembly* (Hong Kong: Lutheran World Service, 1962), p. 3 cited in Clements, *International Significance*, p. 9.

26 See for instance, Peter Gatrell, “Putting Refugees in their Place.” *New Global Studies* 7.1 (2013): 1-24.

27 Megan Bradley et al. “Whither the Refugees? International Organisations and “Solutions” to Displacement, 1921-1960.” *Refugee Survey Quarterly* 41.2 (2022): 159-195.

28 On Humanitarian handicraft see, Claire Barber, Helen Dampier, Rebecca Gill Bertrand Taithe eds. *Humanitarian Handicraft: History, Materiality and Trade* (Manchester: Manchester University Press, forthcoming).

29 Jessica Field, “Consumption in lieu of Membership: Reconfiguring Popular Charitable Action in Post-World War II Britain.” *VOLUNTAS: International Journal of Voluntary and Nonprofit Organizations* 27 (2016): 979-997.

30 Jessica Field, “Serving ‘the Cause’: Cecil Jackson-Cole and the professionalization of charity in post-war Britain.” *Historical Research* 93.260 (2020): 379-397.

31 Vanessa Ogle, “State Rights against Private Capital: The” New International Economic Order” and the Struggle over Aid, Trade, and Foreign Investment, 1962-1981.” *Humanity: An International Journal of Human Rights, Humanitarianism, and Development* 5.2 (2014): 211-234.

32 E.g. Stephen J., Macekura, and Erez Manela, eds. *The Development Century: A Global History*. (Cambridge: Cambridge University Press, 2018).

33 John Silk, “Caring at a distance.” *Ethics, Place and Environment* 1.2 (1998): 165-182.

34 See Bertrand Taithe & Adam Millar, “Living Humanitarian Dreams”, for a full description of these archives.

35 Elizabeth Wilson, *Encounters on the Way* (York: William Session, 1998), p. 174.

36 This is an interest she maintained throughout her life as witness her red diary, c. 1975, Peter Wilson deposit, Humanitarian Archive.

37 Isaac Barnes May, *God-optional Religion in Twentieth-century America: Quakers, Unitarians, Reconstructionist Jews, and the Crisis Over Theism*. New York: Oxford University Press, 2023)

38 Frédéric Lenoir, ‘The Adaptation of Buddhism to the West’, *Diogene*, 187, 47 (1999)3: 100-109; Erik Sand, ed. *Imagining the East: The Early Theosophical Society* (New York: Oxford University Press, 2020).

39 Thich Nhat Hanh, “History of Engaged Buddhism: A Dharma Talk by Thich Nhat Hanh-Hanoi, Vietnam, May 6-7, 2008.” *Human Architecture: Journal of the Sociology of Self-Knowledge* 6,3 (2008): 29-36.

40 Sean Scalmer, “The Labor of Diffusion: the Peace Pledge Union and the Adaptation of the Gandhian Repertoire”, *Mobilization: An International Quarterly*, 7, 3 (2002): 269-286

41 Elizabeth Wilson, ‘Hong Kong Action’ in Pat Arrowsmith ed. *To Asia in Peace*. 121-126, p. 126.

42 Gilles Deleuze and Félix Guattari. *A Thousand Plateaus: Capitalism and Schizophrenia*. (London: Bloomsbury Publishing, 1988).

43 René Grousset, *Sur les Traces du Bouddha (*Paris: Plon, 1929).

44 Fritjof Capra, *The Web of Life: a New Synthesis of Mind and Matter* (London: Harper Collins, 1996).

45 Elizabeth Wilson, “From Darkness”, Types pages, 1998?, Robert Wilson deposit, Humanitarian Archives.

46 Sean Watson, “Chaos of the Body: A Commentary on Fritjof Capra’s The Web of Life.” *Body & Society* 4, no. 3 (1998): 103-114, p. 110-1.

47 Carl Gustav Jung and Suzanne Gieser, *Dream Symbols of the Individuation Process: Notes of C. G. Jung’s Seminars on Wolfgang Pauli’s Dreams* (Princeton: Princeton University Press, 2019). Carl Gustav Jung, *Dream Analysis 1: Notes of the Seminar Given in 1928-30* (London: Routledge, 2013).

48 Michael Ruse, *The Gaia Hypothesis: Science on a Pagan Planet*. (Chicago: University of Chicago Press, 2013).

49 Ilana Feldman, “The Quaker Way: Ethical Labor and Humanitarian Relief.” *American Ethnologist* 34, 4 (2007): 689-705. 690.

50 Wilson, “From Darkness”, s.

51 Albert Schweitzer to Helene Bresslau, June 6 1906, in *The Albert Schweitzer-Helene Bresslau Letters 1902-1912*, eds. Rhena Schweitzer Miller and Gustav Woytt (New York: Syracuse University Press, 2002), 109. ‘I want to have the strength and the health to live my thoughts, so they become life.’

52 Conor Cruise O’Brien, ‘The Schweitzer Legend’, *The New York Review of Books*, August 20 1964; Gerald McKnight, *Verdict on Schweitzer: The Man Behind the Legend of Lambaréné* (New York: John Day Co., 1964); André Audoynaud, *Le docteur Schweitzer et son hôpital à Lambaréné. L’envers d’un mythe* (Paris: l’Harmattan, 2005). Recent African scholarship is rather kinder to Schweitzer: Emane, Augustin. *Albert Schweitzer, une icône africaine*. (Paris: Fayard, 2013).

53 James Brabazon, *Albert Schweitzer: A Biography* (New York: Syracuse University Press, 2000, first edition published 1975), 499.

54 Brabazon, *Albert Schweitzer*, 268.

55 Albert Schweitzer, *Out of My Life and Thought: An Autobiography*, trans. Antje Bultmann Lemke (Maryland: John Hopkins University Press, 1998, [1933]), 156.

56 Albert Schweitzer, *Philosophy of Civilization* (New York: Macmillan Co., 1960), 310-15.

57 Schweitzer, *Out of My Life and Thought*, 227-228.

58 Hilary Blood, ‘Book Review: The Philosophy of Albert Schweitzer by Henry Clark’, *African Affairs*, Vol. 63, No. 253 (Oct., 1964): 301-302.

59 Henry Clark, *The Philosophy of Albert Schweitzer* (Northampton: John Dickens & Co., 1964, first published in the United States as *The Ethical Mysticism of Albert Schweitzer*, 1962), 77.

60 Clark, *The Philosophy of Albert Schweitzer*, 24.

61 Clark, *The Philosophy of Albert Schweitzer*, 167.

62 Albert Schweitzer, Les *Sermons de Lambaréné*, eds Philippe Aubert and Jean-Paul Sorg (Strasbourg: 2002); Albert Schweitzer, *The African Sermons*, edited and translated by Steven E. G. Melamed (New York: Syracuse University Press, 2003). For theological studies and works of Schweitzer, see Philippe Aubert, *Albert Schweitzer: une théologie raisonnable* (Mulhouse: Ralliement Protestant, 1998); Albert Schweitzer and Jean-Paul Sorg (ed.), *Albert Schweitzer, Humanisme et mystique* (Paris: Albin Michel, 1995).

63 Interview with David Hammond, “Caring for the World is the Job of a Lifetime”, *Supplement to the Hudddersfield Daily Examiner*, 11 August, 1998. Robert Wilson deposit, Humanitarian Archive.

64 Interview, ‘Second in our series of personalities profiles’, *Huddersfield Daily Examiner*, c. 1985, deposit Robert Wilson, Humanitarian Archive.

65 L. Ennals “The United Nations Association: The People’s Voice”, *The United Kingdom — The United Nations*. eds E. Jensen, T. Fisher (London: Palgrave Macmillan, 1990), 254-73

66 Lisa Smirl, “Building the Other, Constructing Ourselves: Spatial Dimensions of International Humanitarian Response.” *International Political Sociology* 2.3 (2008): 236-253.

67 David Williams, “Aid as Autobiography.” *Africa* 72, no. 1 (2002): 150-163; Róisín Read,. “Embodying difference: Reading gender in women’s memoirs of humanitarianism.” *Journal of Intervention and Statebuilding* 12.3 (2018): 300-318.

68 Pascale Grellety Bosviel, *Trip Indien: de l’Inde des Hippies à la Création de MSF* (Bordeaux: Elitys, 2012).

69 See Dr Minoru Nomura on Dr Schweitzer, “ ‘Albert Schweitzer’s Way of Thinking’-a Bridge between East and West.” *Universitas* 7, no. 1 (1964): 103-7.

70 This is to take the title of O’Sullivan’s book who in fairness does acknowledge faith-based volunteerism Kevin O’Sullivan,. *The NGO Moment: the Globalisation of Compassion from Biafra to Live Aid*. (Cambridge: Cambridge University Press, 2021).

71 Peter Gatrell, *Free World?: The Campaign to Save the World’s Refugees, 1956-1963*. (Cambridge: Cambridge University Press, 2011).

72 Anna Bocking-Welch, “Imperial legacies and internationalist discourses: British involvement in the United Nations Freedom from Hunger Campaign, 1960-70.” *The Journal of Imperial and Commonwealth History* 40.5 (2012): 879-896.
